# Topology and Phase Transitions: A First Analytical Step towards the Definition of Sufficient Conditions

**DOI:** 10.3390/e23111414

**Published:** 2021-10-27

**Authors:** Loris Di Cairano, Matteo Gori, Marco Pettini

**Affiliations:** 1 Institute of Neuroscience and Medicine INM-9, and Institute for Advanced Simulation IAS-5, Forschungszentrum Jülich, 52428 Jülich, Germany; l.di.cairano@fz-juelich.de; 2 Department of Physics, Faculty of Mathematics, Computer Science and Natural Sciences, Aachen University, 52062 Aachen, Germany; 3 Physics and Materials Science Research Unit, University of Luxembourg, L-1511 Luxembourg, Luxembourg; gori6matteo@gmail.com; 4 Aix-Marseille Univ, CNRS, Université de Toulon, 13288 Marseille, France; 5 CNRS Centre de Physique Théorique UMR7332, 13288 Marseille, France

**Keywords:** phase transitions, differential topology, entropy flow, Weingarten map, Ginzburg-Landau

## Abstract

Different arguments led to supposing that the deep origin of phase transitions has to be identified with suitable topological changes of potential related submanifolds of configuration space of a physical system. An important step forward for this approach was achieved with two theorems stating that, for a wide class of physical systems, phase transitions should necessarily stem from topological changes of energy level submanifolds of the phase space. However, the sufficiency conditions are still a wide open question. In this study, a first important step forward was performed in this direction; in fact, a differential equation was worked out which describes how entropy varies as a function of total energy, and this variation is driven by the total energy dependence of a topology-related quantity of the relevant submanifolds of the phase space. Hence, general conditions can be in principle defined for topology-driven loss of differentiability of the entropy.

## 1. Introduction

At the beginning of the last century, statistical mechanics was introduced to replace the inaccessible knowledge of the dynamics of an N-body system. With the advent of electronic computers, and since the pioneering work of E. Fermi, J. Pasta and S. Ulam, the knowledge of the dynamical behavior of N interacting particles became accessible. Even though the accessible number of degrees of freedom was, and still is, by far smaller than the Avogadro number, so-called molecular dynamics has proven very effective for deriving the macroscopic physical properties of systems, of which the microscopic Hamiltonian dynamics are simulated numerically.

The numerical simulation of microscopic Hamiltonian dynamics can be carried out in the presence of a phase transition. In this case the dynamical approach is equivalent to a microcanonical approach to the study of phase transitions, because the ergodic invariant measure of generic non-integrable Hamiltonian flows is the microcanonical measure. The dynamical approach brings about added value which is absent in the standard study of phase transitions in the canonical ensemble framework, both with analytical methods and numerical Monte Carlo algorithms. In fact, the dynamics of a generic system of N degrees of freedom (particles, classical spins, quasi-particles such as phonons and so on), confined in a finite volume (therein free to move, or defined on a lattice), described by a Hamiltonian

(1) $$H=\frac{1}{2}\sum_{i=1}^{N}p_{i}^{2}+V\left(q_{1},\ldots ,q_{N}\right)\;,$$

or equivalently by the corresponding Lagrangian function

(2) $$L=\frac{1}{2}\sum_{i=1}^{N}{\dot{q}}_{i}^{2}-V\left(q_{1},\ldots ,q_{N}\right)\;,$$

are chaotic. The largest Lyapunov exponent, which quantifies the degree of chaoticity of the dynamics, is a new observable that can be used to characterize phase transitions from a dynamical viewpoint. This can lead rather far by combining the dynamical approach with the natural and effective explanation of the origin of Hamiltonian chaos stemming from the identification of a Hamiltonian flow with a geodesic flow of a suitably defined Riemannian differentiable manifold. This differential geometric framework is defined by endowing configuration space with the non-Euclidean metric of components [1,2,3]

(3) $$g_{ij}=2\left[E-V\left(q\right)\right]\delta_{ij}\;,$$

whence the infinitesimal arc element $ds^{2}=4{\left[E-V\left(q\right)\right]}^{2}dq_{i}\;dq^{i}$; then Newton’s equations are retrieved from the geodesic equations

(4) $$\frac{d^{2}q^{i}}{ds^{2}}+\Gamma_{jk}^{i}\frac{dq^{j}}{ds}\frac{dq^{k}}{ds}=0\;.$$

where $\Gamma_{jk}^{i}$ are the Christoffel connection coefficients of the manifold. In this framework, the degree of instability of the dynamics can be investigated through the Jacobi–Levi-Civita equation for the geodesic deviation vector field J (J locally measures the distance between nearby geodesics), which in a parallel-transported frame reads

(5) $$\frac{d^{2}J^{k}}{ds^{2}}+R_{\;ijr}^{k}\frac{dq^{i}}{ds}J^{j}\frac{dq^{r}}{ds}=0\;.$$

where $R_{\;jkl}^{i}$ are the components of the Riemann curvature tensor. Applied to the configuration space of a physical system, the degree of instability of the phase space trajectories is related to the “landscape” of the curvature of the configuration space manifold, and this can lead—under suitable approximations—to the analytic computation of the largest Lyapunov exponent for high dimensional Hamiltonian flows [4]. In so doing, it was natural to look for some connection between the occurrence of phase transitions and their counterparts in the geometry of the manifolds underlying the flows. This led to discovering that in correspondence with phase transitions, there are peculiar geometrical changes of the mechanical manifolds. Then it turned out that these peculiar geometrical changes were the effects of deeper topological changes of the energy level sets $\Sigma_{E}^{H_{N}}=H_{N}^{-1}\left(E\right)$ and $M_{E}^{H_{N}}={\left\{H_{N}^{-1}\left(\left(-\infty ,E\right]\right)\right\}}_{E\in R}$ [5,6,7,8], where $H_{N}$ is the Hamiltonian function of the physical system. Then this was rigorously ascertained for a few exactly solvable models [4]. Finally, it was found that—for a large class of physical potentials—a phase transition *necessarily* stems from the loss of diffeomorphicity of the $M_{E}^{H_{N}}$, and equivalently, of the $\Sigma_{E}^{H_{N}}$ [9,10,11]. More precisely, it has been proved that diffeomorphicity among the members of the family ${\left\{M_{E}^{H_{N}}\right\}}_{E\in R}$, for any N larger than some $N_{0}$, implies the absence of phase transitions (the necessity theorems have been given a counterexample in reference [12], however, the problem raised by this work has been fixed in [13], and, rigorously, in [14]). This means that the members of the family ${\left\{\Sigma_{E}^{H_{N}}\right\}}_{E<E_{c}}$ are not diffeomorphic to those of the family ${\left\{\Sigma_{H}^{H_{N}}\right\}}_{E>E_{c}}$. As well, the members of the family ${\left\{M_{E}^{H_{N}}\right\}}_{E<E_{c}}$ are not diffeomorphic to those of ${\left\{M_{E}^{H_{N}}\right\}}_{E>E_{c}}$. If $E_{c}$ stands for the critical value of the Hamiltonian function at the transition point. Hence, the starting of a topological theory of phase transitions that goes beyond the existing theories on this topic [4]—namely, the Yang-Lee theory [15,16] and the Dobrushin–Lanford–Ruelle theory [17]—requires the limit N→∞ to account for the loss of analyticity of thermodynamic observables; but the study of transitional phenomena in finite N systems (with N extremely smaller than the Avogadro number) is particularly relevant in many other contemporary problems [18], for instance, those related to polymers’ thermodynamics and biophysics [19,20,21], Bose–Einstein condensation and Dicke’s superradiance in microlasers, superconductive transitions in small metallic objects, just to quote some example.

On the other hand, the Landau theory, which relates phase transitions with the symmetry-breaking phenomenon, is not an all-encompassing theory because there are many systems undergoing phase transitions in the absence of an order parameter, and thus in the absence of symmetry-breaking [22,23]. Therefore, looking for generalizations of the existing theories is a well motivated and timely purpose.

Now, an explicit link between thermodynamics and topology is provided by the following exact formula:

$$S_{N}\left(E\right)=\left(k_{B}/N\right)\log\left[\int_{M_{E}^{H_{N}}}\;d^{N}q\;d^{N}p\right]$$

(6) $$=\frac{k_{B}}{N}\log\left[vol\left[M_{E}^{H_{N}}\setminus \overset{N(E)}{\underset{i=1}{⋃}}\Gamma \left(x_{c}^{\left(i\right)}\right)\right]\;+\sum_{i=0}^{N}w_{i}\;\mu_{i}\left(M_{E}^{H_{N}}\right)+R\right],$$

where $S_{N}$ is the entropy, E is the total energy per degree of freedom and the $\mu_{i}\left(M_{E}^{H_{N}}\right)$ are the Morse indexes (in one-to-one correspondence with topology changes) of the submanifolds $M_{E}^{H_{N}}={\left\{H_{N}^{-1}\left(\left(-\infty ,E\right]\right)\right\}}_{E\in R}$ of the phase space. The first term of Equation (6) in square brackets is the result of the excision of certain neighborhoods of the critical points of the Hamiltonian function from $M_{E}^{H_{N}}$; the second term is a weighed sum of the Morse indexes; and the third term is a smooth function of N and E. It is evident that sharp changes in the Hamiltonian function pattern of at least some of the $\mu_{i}\left(M_{E}^{H_{N}}\right)$ (thus of the way topology changes with E) could affect $S_{N}\left(E\right)$ and its derivatives. In what follows, the development of a fundamental analytical tool is shown, which was made to pave the way to future investigations about the different kinds of topology changes that can entail a phase transition, thereby providing the theory with *sufficiency conditions*.

## 2. Entropy Flow

For a long time, it has been well known [24,25,26] that the statistical description of the phase transitions (PTs) of an autonomous Hamiltonian system—which consists of defining suitable integration measures—can be re-phrased and re-interpreted within a geometric/topological framework—where one studies which geometric-topological changes in phase space can give rise to a PT. In the microcanonical ensemble, the relation between such two descriptions can be realized by working with the entropy function which is the fundamental thermodynamic potential. In fact, being defined as a function of energy, S(E), all the thermodynamic observables can be deduced, through suitable combinations of its derivatives with respect to the energy. Conceptually, given an Hamiltonian function H:Λ→R defined on the phase space, $\Lambda \subset R^{N}\times R^{N}$, and by adopting Boltzmann’s entropy definition, the entropy of the system at a given energy, E, is

(7) $$S\left(E\right)=\log\left(vol^{\chi }\left(\Sigma_{E}^{H}\right)\right),$$

with

(8) $$vol^{\chi }\left(\Sigma_{E}^{H}\right)=\int_{\Sigma_{E}^{H}}\chi \;d\sigma_{\Sigma_{E}^{H}},$$

where $d\sigma_{\Sigma_{E}^{H}}$ is the Euclidean volume measure induced on $\Sigma_{E}^{H}$ and with the energy level set

(9) $$\Sigma_{E}^{H}=H^{-1}\left(E\right)=\left\{x\in \Lambda \;|\;H\left(x\right)=E\right\}\subset R^{N}\times R^{N}.$$

The function χ reminds us that one can actually consider a weighted volume measure, as we discuss in a moment, for physical reasons.

In order to advance, the crucial point is that to provide a definition for the function χ in Equation (8), which in turn, will allow us to explicitly define $vol^{\chi }$. It is worth noting that the definition of an integration measure is a matter of choice. Actually, from a purely mathematical viewpoint, any energy level set, $\Sigma_{E}^{H}$, being viewed as a topological space, can be equipped with a Borel measure so as to be *compatible* with the topology of any energy level set.

On the basis of statistical mechanics assumptions, we can restrict the range of possibilities. In fact, a *natural* choice is to consider a measure that, by virtue of Liouville theorem, is invariant with respect to the Hamiltonian flow—namely, it has to be preserved by the dynamics. This can be done by defining, on a given energy level set, $\Sigma_{E}^{H}$, the following function:

(10) $$\chi =\frac{1}{∥\nabla H∥},$$

where ∥·∥ is the Euclidean norm in Λ and $\nabla =\{\partial_{p_{1}},\ldots ,\partial_{p_{N}},\partial_{q^{1}},\ldots ,\partial_{q^{N}}\}$ is the gradient operator acting on phase-space-valued functions. Therefore, for physical reasons, rather then choosing an integration measure which exactly produces the geometric volume, i.e., χ=1 so that $vol^{\chi =1}\equiv vol$, one is led to consider, a volume measure weighted by the function ${∥\nabla H∥}^{-1}$. Thus, from definition (10), the weighted volume (8) reads:

(11) $$vol^{\chi }\left(\Sigma_{E}^{H}\right)=\int_{\Sigma_{E}^{H}}\frac{d\sigma_{\Sigma_{E}^{H}}}{∥\nabla H∥},$$

and the entropy

(12) $$S\left(E\right)=\log\left(\int_{\Sigma_{E}^{H}}\frac{d\sigma_{\Sigma_{E}^{H}}}{∥\nabla H∥}\right).$$

In order to better understand our aim, a remark is now in order. For the sake of simplicity, let us discuss in terms of the first derivative of S(E), but obviously, it can be extended to higher order derivatives.

Roughly speaking, the differentiation under the integral sign in Equation (12) involves derivatives of the induced measure $d\sigma_{\Sigma_{E}^{H}}$ and of ${∥\nabla H∥}^{-1}$. Moreover, the former evidently contains the information about the geometry of the energy level set $\Sigma_{E}^{H}$, whereas the latter adds a further contribution to the entropy variation, but it cannot be directly traced back to any geometric origin.

Therefore, a question naturally arises: what is the role played by the geometry of the $\Sigma_{E}^{H}$ in a PT? In other words, how relevant is the geometric contribution at the ground of a PT?

Before answering these questions, one should firstly wonder: is it legitimate to drop out the ${∥\nabla H∥}^{-1}$ term? It is worth noting that—at least in systems with small numbers of degrees of freedom—there exists a positive answer to the last question, as has been shown in [27,28]. In this setting, i.e., when N is relatively small, a more appropriate entropy definition is given by

(13) $$S\left(E\right)=\log\int_{\Sigma_{E}^{H}}d\sigma_{\Sigma_{E}^{H}},$$

which consists of setting χ=1 in Equation (8) so that the weighted volume reduces to the geometric one:

(14) $$vol\left(\Sigma_{E}^{H}\right)=\int_{\Sigma_{E}^{H}}d\sigma_{\Sigma_{E}^{H}}.$$

Clearly, for systems with large numbers of degrees of freedom, Boltzmann entropy in Equation (12) is the correct one. However, as a first step in compliance with the aim of our present work, we begin by using the entropy defined in Equation (13).

### 2.1. Introduction to the Geometric Approach

Let us consider an interval $S=(E_{0},E_{1})$ in an energy domain such that the Hamiltonian has no critical points; i.e.,

$$\begin{array}{cc} dH{|}_{x}\neq 0 & \forall x\in S. \end{array}$$

This allows one to introduce a regular foliation of the the phase space region defined as

(15) $$\Lambda =\underset{E\in S}{⋃}\;\Sigma_{E}^{H}.$$

Of course, a natural symplectic structure on phase space does not carry any Riemannian geometric structure. Moreover, we assume that any energy level set $\Sigma_{E}^{H}$ is equipped with a metric tensor $h_{E}$, resulting in a family of Riemannian metrics ${\left\{h_{E}\right\}}_{E\in S}$. In so doing, a Riemannian metric is defined on the whole phase space as [29]

(16) $$g_{\Lambda }=\frac{dE}{∥\nabla H∥}⊗\frac{dE}{∥\nabla H∥}+h_{E};$$

hence, the volume measure on the phase space

(17) $$d\mu^{g_{\Lambda }}=\frac{\sqrt{\det\;h_{E}}}{∥\nabla H∥}\;dy^{1}\ldots dy^{n-1}dE,$$

where n=2N is the dimension of the phase space, and ${\left\{y_{E}^{i}\right\}}_{i=1}^{n-1}$ is the system of coordinates on $\Sigma_{E}^{H}$. Thus, the induced measure on the energy level which defines the entropy in Equation (12) corresponds to:

(18) $$d\mu^{g_{\Lambda }}{|}_{\Sigma_{E}^{H}}=\frac{d\sigma_{\Sigma_{E}^{H}}}{∥\nabla H∥}=\frac{\sqrt{\det\;h_{E}\left(y\right)}}{∥\nabla H∥}\;dy_{E}^{1}\ldots dy_{E}^{n-1}.$$

A remark is in order. In writing the Riemannian metric tensor in Equation (16), we have tacitly introduced a curvilinear coordinate system on Λ, i.e., $\left\{u^{0},u^{1},\ldots ,u^{n-1}\right\}$, such that $u^{0}=E$ and $u^{i}=y^{i}$. This set of coordinates gives rise to a vector basis together with its dual, respectively,

(19) $$\begin{array}{cc} \{\partial_{u^{0}},\partial_{u^{1}},\ldots ,\partial_{u^{n-1}}\}, & \{du^{0},du^{1},\ldots ,du^{n-1}\}, \end{array}$$

such that

(20) $$\begin{array}{cc} du^{\alpha }\left(\partial_{u^{\beta }}\right)=\delta_{\beta }^{\alpha }, & \alpha ,\beta =0,1,\ldots ,n-1. \end{array}$$

In order to better understand the role of $\partial_{u^{0}}$, we introduce the unit normal vector field to the energy level sets

(21) $$\nu =\frac{\nabla H}{∥\nabla H∥},$$

and since g(ν,ν)=1, we necessarily have $du^{0}\left(\nu \right)=\chi^{-1}$. Moreover, $g(\nu ,\partial_{u^{i}})=0$ for every i=1,…,n−1; thus, we can write $\partial_{u^{0}}=f\;\nu$ where f is an unknown function that, through the condition (20), produces f=χ. This means that the basis vector $\partial_{u^{0}}$ actually is not normalized and $∥\partial_{u^{0}}∥=g_{\Lambda }{\left(\partial_{u^{0}},\partial_{u^{0}}\right)}^{1/2}=\chi$. In fact, we note that the reference frame and its dual given in Equation (19) represent a moving frame.

Therefore, $\partial_{u^{0}}$ is the normal vector field which generates diffeomorphisms between energy level sets. Roughly speaking, the introduction of a Hamiltonian function H:Λ→R provides a parametrization $H\left(\Sigma_{E}^{H}\right)=E\equiv u^{0}$, which, in turn, gives rise to a covering of the phase space in terms of energy level sets. As a consequence, any variation of the energy value, $E\to E^{\prime }$, is associated with a diffeomorphism $\Sigma_{E}^{H}\to \Sigma_{E^{\prime }}^{H}$ generated by the flow of the vector field $\partial_{u^{0}}=\chi \nu$ [29,30].

In the end, such a fact implies that the practical and correct way for differentiating the entropy function (13) with respect to the energy is by means of $\partial_{u^{0}}$, and it is associated with the Lie operator $L_{\partial_{u^{0}}}$, which clearly differs from that defined by the unit normal vector (21) denoted with $L_{\nu }$.

We stress that, although the above-mentioned differentiation is the rigorous one, we actually adopt a different parameterization, e, based on the unit normal vector ν and such that de(ν)=1. In this way, we give the following definition (different than the definition in Equation (16)) of the metric tensor with respect to the energy-like variable:

(22) $$g_{\Lambda }=de⊗de+h_{E}.$$

Such a parametrization can be geometrically interpreted as a normal deformation of the energy level set as the previous one (the vector $\partial_{u^{0}}$ is normal to $\Sigma_{H}^{E}$ and ν), but we have an equidistant motion of energy level sets [31] since, in Equation (22), the tensor de⊗de is not multiplied by any function.

### 2.2. Entropy Flow Equation

We now show the main result of this paper, namely—based on the entropy definition (13)—one can define a nonlinear differential equation describing the entropy flow as a function of energy. We then recognize that the forcing term in this equation depends on well identifiable geometric features such as scalar curvature and Weingarten operator.

In order to obtain the equation for the entropy flow, let us rewrite Equation (13) as

(23) $$S\left(e\right)=\log\left(vol(\Sigma_{e}^{H})\right)\equiv \log\int_{\Sigma_{e}^{H}}d\sigma_{\Sigma_{e}^{H}};$$

then, let us compute the first and second order e-derivatives. We have:

(24) $$\partial_{e}S\left(e\right)=\frac{\partial_{e}vol\left(\Sigma_{e}^{H}\right)}{vol(\Sigma_{e}^{H})},$$

(25) $$\partial_{e}^{2}S\left(e\right)=\frac{\partial_{e}^{2}vol\left(\Sigma_{e}^{H}\right)}{vol(\Sigma_{e}^{H})}-{\left(\frac{\partial_{e}vol\left(\Sigma_{e}^{H}\right)}{vol(\Sigma_{e}^{H})}\right)}^{2}.$$

By substituting the first equation into the second term in the right-hand side of the second equation, one finds

(26) $$\partial_{e}^{2}S\left(e\right)+{\left(\partial_{e}S\left(e\right)\right)}^{2}=\frac{\partial_{e}^{2}vol\left(\Sigma_{e}^{H}\right)}{vol(\Sigma_{e}^{H})}.$$

The equation above is a Riccati differential equation [32] describing the flow of entropy driven just by geometry, as we will see later on. Meanwhile, we anticipate that the geometric contribution is contained in the non-homogeneous term which is proportional to the second derivative of the volume; therefore, it is necessary to explicitly compute the second volume variation.

#### 2.2.1. First Variation of Volume

The first volume variation formula can be obtained by computing the first e-derivative of the volume measure:

(27) $$\partial_{e}vol\left(\Sigma_{e}^{H}\right).$$

This quantity can be explicitly written, noting that the Lie derivative with respect to the unit normal vector ν applied to the induced metric $h_{e}$ is twice the second fundamental form II [29,31]:

(28) $$L_{\nu }h_{e}=2\;I\;\;I.$$

Thus, Equation (27) becomes:

(29) $$\partial_{e}vol\left(\Sigma_{e}^{H}\right)=\int_{\Sigma_{e}^{H}}L_{\nu }\left(d\sigma_{\Sigma_{e}^{H}}\right)=\int_{\Sigma_{e}^{H}}Tr\left[I\;\;I\right]\;d\sigma_{\Sigma_{e}^{H}},$$

since [4,33]

$$L_{\nu }\sqrt{\det h_{e}}=\frac{\left(\det h_{e}\right)}{2\sqrt{\det h_{e}}}C\left[h_{e}^{-1}L_{\nu }h_{e}\right]=\sqrt{\det h_{e}}Tr\left[I\;\;I\right],$$

where C is the total contraction operator which yields the trace of its argument.

The Weingarten (or shape) operator of an immersed manifold is defined on $\Sigma_{e}^{H}$ as [29,31,33]

(30) $$W_{\nu }\left(X\right)=\nabla_{X}\nu ,$$

for any X which lies on the tangent space to the energy level set $\Sigma_{e}^{H}$. The trace of the second fundamental form coincides with the trace of the Weingarten operator

(31) $$Tr\left[I\;\;I\right]=\sum_{i=1}^{n-1}I\;\;I\left(a_{i},a_{i}\right)=\sum_{i=1}^{n-1}h_{e}\left(W_{\nu }\left(a_{i}\right),a_{i}\right)=\sum_{i=1}^{n-1}h_{e}\left(\nabla_{a_{i}}\nu ,a_{i}\right)\equiv Tr\left[W_{\nu }\right],$$

where ${\left\{a_{i}\right\}}_{i=1}^{n-1}$ is an orthonormal basis on $\Sigma_{e}^{H}$.

Therefore, one has

(32) $$\frac{\partial vol(\Sigma_{e}^{H})}{\partial e}\left(e\right)=\int_{\Sigma_{e}^{H}}Tr\left[W_{\nu }\right]\;d\sigma_{\Sigma_{e}^{H}}.$$

#### 2.2.2. Second Variation of Volume

The second volume variation formula can be obtained by deriving once more Equation (32) with respect to the e-variable, and it is

(33) $$\frac{\partial^{2}vol\left(\Sigma_{e}^{H}\right)}{\partial e^{2}}\left(e\right)=\int_{\Sigma_{e}^{H}}L_{\nu }\left\{Tr\left[W_{\nu }\right]\;d\sigma_{\Sigma_{e}^{H}}\right\}=\int_{\Sigma_{e}^{H}}\left\{Tr\left[\nabla_{\nu }W_{\nu }\right]+{\left(Tr\left[W_{\nu }\right]\right)}^{2}\right\}d\sigma_{\Sigma_{e}^{H}}.$$

Then, by taking the trace of the Riccati equation for the Weingarten operator [29]:

(34) $$Tr\left[\nabla_{\nu }W_{\nu }\right]=-Tr\left[W_{\nu }^{2}\right]-Ric\left(\nu ,\nu \right),$$

where Ric(ν,ν) is the Ricci curvature along the vector field ν which identically vanishes in $R^{n}$, and by plugging it in Equation (33), one gets the second variation formula for the volume

(35) $$\frac{\partial^{2}vol\left(\Sigma_{e}^{H}\right)}{\partial e^{2}}\left(e\right)=\int_{\Sigma_{e}^{H}}\left\{Tr{\left[W_{\nu }\right]}^{2}-Tr\left[W_{\nu }^{2}\right]\right\}d\sigma_{\Sigma_{e}^{H}}.$$

The equation above obtained contains the essence of our final result, and therefore, a remark is worth making. The second variation of volume (acceleration) is driven by a particular combination of the trace of Weingarten operator which is related to the scalar curvature of the energy level set. In order to see it, let us consider the following decomposition (see decomposition in Section 2.3 of [31])

(36) $$R\left(\Lambda \right)=2\;Ric\left(\nu ,\nu \right)+R\left(\Sigma_{e}^{H}\right)+Tr\left[W_{\nu }^{2}\right]-Tr{\left[W_{\nu }\right]}^{2},$$

where R(Λ) and $R(\Sigma_{e}^{H})$, respectively, are the scalar curvatures of phase space Λ (ambient manifold) and of the energy level set, and since the ambient space Λ is Euclidean, we have

(37) $$\begin{array}{cc} Ric(\nu ,\nu )=0, & R(\Lambda )=0, \end{array}$$

which implies

(38) $$R\left(\Sigma_{e}^{H}\right)=Tr{\left[W_{\nu }\right]}^{2}-Tr\left[W_{\nu }^{2}\right].$$

Hence, the second variation formula of volume can be recast into a more intuitive fashion:

(39) $$\frac{d^{2}vol\left(\Sigma_{e}^{H}\right)}{de^{2}}\left(e\right)=\int_{\Sigma_{e}^{H}}R\left(\Sigma_{e}^{H}\right)\;d\sigma_{\Sigma_{e}^{H}}.$$

#### 2.2.3. Entropy Flow

By substituting Equation (39) into Equation (26), the entropy flow is described by

(40) $$\partial_{e}^{2}S\left(e\right)+{\left(\partial_{e}S\left(e\right)\right)}^{2}=\int_{\Sigma_{e}^{H}}R\left(\Sigma_{e}^{H}\right)\;d\eta_{\Sigma_{e}^{H}},$$

where the normalized measure

(41) $$d\eta_{\Sigma_{e}^{H}}=\frac{d\sigma_{\Sigma_{e}^{H}}}{vol(\Sigma_{e}^{H})}.$$

has been introduced.

An appropriate definition of Equation (40) would be that it is a *geometric entropy flow equation*. In fact, the choice of the energy-like parametrization e associated with the Lie derivative $L_{\nu }$ entails that the second derivative of the volume depends only on the way the geometry of the energy level sets varies with the energy-like parameter e.

This can be observed by acting twice with the Lie derivative $L_{\partial_{E}}=\chi L_{\nu }$ on the volume, that is,

(42) $$\partial_{E}^{2}vol\left(\Sigma_{E}^{H}\right)=\int_{\Sigma_{E}^{H}}\chi \left(\nabla_{\nu }\chi \right)\;Tr\left[W_{\nu }\right]d\sigma_{\Sigma_{E}^{H}}+\int_{\Sigma_{E}^{H}}\chi^{2}\;R\left(\Sigma_{E}^{H}\right)d\sigma_{\Sigma_{E}^{H}},$$

and comparing the outcome with Equation (39). Hence, it is clear by inspection that geometric factors such as the trace of the Weingarten operator and scalar curvature are multiplied by factors as $\chi \nabla_{\nu }\chi$ and $\chi^{2}$, respectively, which cannot be given a geometric interpretation.

Moreover, in addition to the assumptions introduced in Section 2.1, we have also assumed that the first and second order derivatives (see Equations (24) and (25)) of the entropy function (23) represent a closed system of differential equations that can be reduced to Equation (40). In fact, one can recognize in the second term of the right-hand side of Equation (25), i.e., $\partial_{e}vol\left(\Sigma_{E}^{H}\right)/vol\left(\Sigma_{E}^{H}\right)$, the first derivative of S(e) given in Equation (24). Then, one assumes that the term $\partial_{e}^{2}vol\left(\Sigma_{E}^{H}\right)/vol\left(\Sigma_{E}^{H}\right)$ is not related to any combination of the derivatives of the entropy, and in fact, one can show that it coincides with the normalized integral of the scalar curvature of the energy level sets. Therefore, the system of equations is shown to be closed since the previous term is just of geometric meaning.

In principle, one could include in the system (24) and (25) higher order derivatives of entropy to obtain a differential equation for the entropy equivalent to Equation (40). For instance, let us consider the third order derivative

(43) $$\begin{array}{ccc} \partial_{e}S\left(e\right) & = & \frac{\partial_{e}vol\left(\Sigma_{e}^{H}\right)}{vol(\Sigma_{e}^{H})}, \end{array}$$

(44) $$\partial_{e}^{2}S\left(e\right)=\frac{\partial_{e}^{2}vol\left(\Sigma_{e}^{H}\right)}{vol(\Sigma_{e}^{H})}-{\left(\frac{\partial_{e}vol\left(\Sigma_{e}^{H}\right)}{vol(\Sigma_{e}^{H})}\right)}^{2},$$

(45) $$\partial_{e}^{3}S\left(e\right)=\frac{\partial^{3}vol\left(\Sigma_{e}^{H}\right)}{vol(\Sigma_{e}^{H})}-3\frac{\partial_{e}^{2}vol\left(\Sigma_{e}^{H}\right)}{vol(\Sigma_{e}^{H})}\frac{\partial_{e}vol\left(\Sigma_{e}^{H}\right)}{vol(\Sigma_{e}^{H})}+2{\left(\frac{\partial_{e}vol\left(\Sigma_{e}^{H}\right)}{vol(\Sigma_{e}^{H})}\right)}^{3}.$$

Now, by substituting in Equation (45), the first and second derivatives, we get

(46) $$\partial_{e}^{3}S\left(e\right)+3\partial_{e}^{2}S\left(e\right)\partial_{e}S\left(e\right)+{\left(\partial_{e}S\left(e\right)\right)}^{3}=\frac{\partial^{3}vol\left(\Sigma_{e}^{H}\right)}{vol(\Sigma_{e}^{H})}.$$

Here, the right-hand side can be interpreted again as a term of purely geometric meaning, and in fact [29]

(47) $$\begin{array}{cc} \partial^{3}vol\left(\Sigma_{e}^{H}\right) & =\frac{\partial }{\partial e}\int_{\Sigma_{e}^{H}}R\left(\Sigma_{e}^{H}\right)\;d\sigma_{\Sigma_{e}^{H}}=\int_{\Sigma_{e}^{H}}\left(\partial_{e}R\left(\Sigma_{e}^{H}\right)+R\left(\Sigma_{e}^{H}\right)\;Tr\left[I\;\;I\right]\right)\;d\sigma_{\Sigma_{e}^{H}}\\ & =-2\int_{\Sigma_{e}^{H}}Tr\left[G\cdot W_{\nu }\right]\;d\sigma_{\Sigma_{e}^{H}}, \end{array}$$

where $G=Ric\left(\Sigma_{e}^{H}\right)-\frac{1}{2}h_{e}\;R\left(\Sigma_{e}^{H}\right)$ is the contravariant Einstein tensor, $Ric(\Sigma_{e}^{H})$ is the Ricci curvature tensor of the energy level set whereas $G\cdot W_{\nu }$, in the last equality, stands for the matrix product, that is, ${\left(G\cdot W_{\nu }\right)}_{ij}=G_{ik}{\left(W_{\nu }\right)}_{j}^{k}$.

Therefore, Equation (46) together with (47) defines a differential equation for the entropy function which contains the same thermodynamic information contained in Equation (40). Alternatively, one could define a sort of *geometric temperature* function defined by $T\left(e\right):=\partial_{e}S\left(e\right)$, and Equation (46) becomes an equation for the geometric temperature:

(48) $$\partial_{e}^{2}T\left(e\right)+3\left(\partial_{e}T\left(e\right)\right)T\left(e\right)+T{\left(e\right)}^{3}=-2\int_{\Sigma_{e}^{H}}Tr\left[G\;W_{\nu }\right]\;d\sigma_{\Sigma_{e}^{H}}.$$

It is worth mentioning that in reference [34], similar investigations were proposed concerning the relation between geometric and thermodynamic quantities and their behavior in the presence of a PT; in this context, the derivatives of the entropy are treated as *observables*, such as temperature, specific heat and the second order derivative of the entropy. Contrarily, our present approach treats S as an unknown function which can be determined by solving Equation (40).

An equivalent and very interesting version of the entropy flow Equation (40) can be given in the form of a harmonic oscillator-like equation. In fact, by introducing a function Y, which plays the role of a volume, such that

(49) $$S\left(e\right)=\log\left(\frac{Y(e)}{Y(e_{0})}\right),$$

where $Y(e_{0})$ is related to the volume value corresponding to the lowest accessible energy value; and by replacing this relation into Equation (40), we get

(50) $$\partial_{e}^{2}Y\left(e\right)-\left(\int_{\Sigma_{e}^{H}}R\left(\Sigma_{e}^{H}\right)\;d\mu_{\Sigma_{e}}\right)Y\left(e\right)=0.$$

This equation already allows one to infer some interesting properties of the energy level sets, at least in the present framework of energy-like parametrization. In practice, the total scalar curvature needs to be positive in order to be compatible with a physically meaningful entropy function.

With the help of the Hölder inequality for integrals, we have

(51) $$\int_{\Sigma_{e}^{H}}R\left(\Sigma_{e}^{H}\right)\;d\sigma_{\Sigma_{e}^{H}}\leq \int_{\Sigma_{e}^{H}}\left|R\left(\Sigma_{e}^{H}\right)\right|\;d\sigma_{\Sigma_{e}^{H}}\leq {\left[\int_{\Sigma_{e}^{H}}\{|R\left(\Sigma_{e}^{H}\right){\left|\right\}}^{n/2}d\sigma_{\Sigma_{e}^{H}}\right]}^{2/n}{\left[\int_{\Sigma_{e}^{H}}d\sigma_{\Sigma_{e}^{H}}\right]}^{1/(1-2/n)},$$

whence, at large n,

(52) $$\int_{\Sigma_{e}^{H}}R\left(\Sigma_{e}^{H}\right)\;d\mu_{\Sigma_{e}^{H}}=\int_{\Sigma_{e}^{H}}R\left(\Sigma_{e}^{H}\right)\;d\sigma_{\Sigma_{e}^{H}}{\left[\int_{\Sigma_{e}^{H}}d\sigma_{\Sigma_{e}^{H}}\right]}^{-1}={\left[\int_{\Sigma_{e}^{H}}\{|R\left(\Sigma_{e}^{H}\right){\left|\right\}}^{n/2}d\sigma_{\Sigma_{e}^{H}}\right]}^{2/n}-r\left(e\right),$$

where r(e) is a positive remainder function. The scalar curvature is here given by

$$R\left(\Sigma_{e}^{H}\right)=\sum_{i<j}\kappa_{i}\kappa_{j},$$

where the $\kappa_{i}$ are principal curvatures of $\Sigma_{e}^{H}$. Using the expression of a multinomial expansion

(53) $${\left(x_{1}+\cdots +x_{\nu }\right)}^{\nu }=\sum_{{}_{\left\{n_{i}\right\},\sum n_{k}=\nu }}\frac{\nu !}{n_{1}!\cdots n_{\nu }!}\cdot x_{1}^{n_{1}}\cdots x_{\nu }^{n_{\nu }}\;\;,$$

we can write

(54) $$|R\left(\Sigma_{e}^{H}\right){|}^{n/2}=n\left(n-1\right)\left(\frac{n}{2}\right)!\left|\kappa_{1}\kappa_{2}\cdots \kappa_{n}\right|+R\left(e\right),$$

where R(e) contains all the terms of the multinomial expansion (53) but that one with $n_{1}=n_{2}=\cdots =n_{\nu }=1$. Then, since $|\kappa_{1}\kappa_{2}\cdots \kappa_{n}\left|=\right|K_{G}|$, where $|K_{G}|$ is the Gauss–Kronecker curvature, by resorting to the Chern–Lashof theorem [35]:

(55) $$\int_{\Sigma_{e}^{H}}d\sigma_{\Sigma_{e}^{H}}\;\left|K_{G}\right|=\frac{1}{2}\mathrm{vol}\left(S_{1}^{n-1}\right)\sum_{i=0}^{n}\mu_{i}\left(\Sigma_{e}^{H}\right)\;,$$

where $\mu_{i}\left(\Sigma_{e}^{H}\right)$ are the Morse indexes of $\Sigma_{e}^{H}$, immersed in the Euclidean space $R^{n}$, $S^{n}$ is an n-dimensional sphere of unit radius and $d\sigma_{\Sigma_{e}^{H}}$ is the measure on $\Sigma_{e}^{H}$. Thus, we can rewrite Equation (50) as

(56) $$\partial_{e}^{2}Y\left(e\right)-\left\{n\left(n-1\right)\left(\frac{n}{2}\right)!\;\mathrm{vol}\left(S_{1}^{n-1}\right)\sum_{i=0}^{n}\mu_{i}\left(\Sigma_{e}^{H}\right)+\int_{\Sigma_{e}^{H}}R\left(e\right)\;d\sigma_{\Sigma_{e}^{H}}-r\left(e\right)\right\}Y\left(e\right)=0\;.$$

This equation represents a first interesting step toward the definition of sufficient topological conditions to entail a PT. If the remainder functions R(e) and r(e) are smooth, the topological problem is now converted into a problem in real analysis. In other words, by relating the differentiability class of the function Y(e) (and thus of the entropy S(e)) to the energy variation property of the topological term in curly brackets, the mentioned *sufficient* conditions can be obtained. Of course, this equation requires further clarifications because the Morse indexes are integers so that the topological term is not a differentiable function, and the remainder functions R(e) and r(e) have to “round its edges”. All this will be the subject of further investigations. This first step paves the way to constructive definitions of classes of topological changes of the energy level sets that can entail a PT.

## 3. A Consistency Check

In this section, we test the entropy flow Equation (40) in case of two minimalistic systems: harmonic oscillator and Ginzburg–Landau-like potentials.

### 3.1. Harmonic Oscillators and Ginzburg–Landau-Like Potential

We consider a physical system composed by N particles and described by a Ginzburg–Landau-like potential (GL) function

(57) $$V_{GL}\left(q\right)=-\frac{\alpha }{2}\sum_{i=1}^{N}{\left(q^{i}\right)}^{2}+\frac{\beta }{4}{\left(\sum_{i=1}^{N}{\left(q^{i}\right)}^{2}\right)}^{2},$$

where the set of coordinates $q=\{q^{1},\ldots ,q^{N}\}$ has been introduced with $q^{i}\in R$ for every i∈[1,N] and $\alpha ,\;\beta \in R^{+}$.

We note that the harmonic oscillators (HOs) system can be obtained from the potential (57) by setting

(58) α→−α,β→0,

and one gets:

(59) $$V_{HO}\left(q\right)=\frac{\alpha }{2}\sum_{i=1}^{N}{\left(q^{i}\right)}^{2}.$$

For the Hamiltonian function, we used the same notation adopted for the potential: namely, we denote with $H_{GL}$ and $H_{HO}$ the GL and HO Hamiltonian functions, respectively.

By denoting with $p=\{p_{1},\ldots ,p_{N}\}$ the set of momenta, we can define an energy level set to be:

(60) $$\Sigma_{E}^{H_{GL}}=\left\{\left(p,q\right)\in \Lambda \;|\;\sum_{i=1}^{N}\frac{p_{i}^{2}}{2}-\frac{\alpha }{2}\sum_{i=1}^{N}{\left(q^{i}\right)}^{2}+\frac{\beta }{4}{\left(\sum_{i=1}^{N}{\left(q^{i}\right)}^{2}\right)}^{2}=E_{GL}\right\}.$$

Depending on the value of E, the energy level sets show a change in their topology. In fact, by defining the *order parameter*

(61) $$r^{2}=\sum_{i=1}^{N}{\left(q^{i}\right)}^{2},$$

together with the squared norm of the total momentum:

(62) $$P^{2}=\sum_{i=1}^{N}p_{i}^{2},$$

the Hamiltonian function whose potential is the one in Equation (57) is rewritten as

(63) $$H_{GL}\left(P,r\right)=\frac{P^{2}}{2}-\frac{\alpha }{2}r^{2}+\frac{\beta }{4}r^{4},$$

and it admits three classes of stationary points given by the condition $\nabla H_{GL}=0$—namely:

(64) $$\begin{array}{cc} \frac{\partial H_{GL}}{\partial P}=P=0, & \frac{\partial H_{GL}}{\partial r}=\left(-\alpha +\beta r^{2}\right)r=0. \end{array}$$

This implies that

(65) $$r_{m}^{\pm }=\pm \sqrt{\frac{\alpha }{\beta }}\;\mathrm{and}\;P_{m}=0,\;r_{M}=0\;\mathrm{and}\;P_{M}=0,$$

where the subscript m stands for minima and M for the maximum; and finally, in terms of particle coordinates, we have

(66) $${\left(\sum_{i=1}^{N}{\left(q_{\pm }^{i}\right)}^{2}\right)}^{1/2}=\pm \sqrt{\frac{\alpha }{\beta }}\;\mathrm{and}\;p_{j}=0,\;q^{i}=0\;\mathrm{and}\;p_{j}=0,\;\;\forall \;j\in \left[1,N\right].$$

Therefore, the energy level set are defined by

(67) $$H_{GL}\left(P,r\right)=\frac{P}{2}-\frac{\alpha }{2}r^{2}+\frac{\beta }{4}r^{4}=E_{GL}.$$

Not all the energy values $E_{GL}$ are associated with an accessible energy level set. In fact:

(68) $$\forall \;E_{GL}<H_{GL}\left(P_{m},r_{m}^{\pm }\right)=-\frac{\alpha^{2}}{4\beta }\Rightarrow \Sigma_{E}^{H_{GL}}=\emptyset \;.$$

Therefore, the lowest energy value is $E_{GL}^{m}=-\alpha^{2}/4\beta$ and the accessible level sets are defined by the following range of energies:

(69) $$E\in [-\alpha^{2}/4\beta ,\infty ).$$

Thus, the energy level sets corresponding to the energy values $H\left(P_{m},r_{m}^{\pm }\right)\leq E_{GL}<H_{GL}\left(P_{M},r_{M}\right)=0$ are homeomorphic to two disjoint hyperspheres:

(70) $$\Sigma_{E}^{H_{GL}}≃S^{n-1}\cup S^{n-1},$$

As $E_{GL}=0$, the energy level set $\Sigma_{0}^{H_{GL}}$ is homeomorphic to the one-point-union of the two previous hyperspheres, and this can be written through the wedge sum:

(71) $$\Sigma_{0}^{H_{GL}}≃S^{n-1}\wedge S^{n-1}=\left(S^{n-1}\cup S^{n-1}\right)/∼,$$

where ∼ is the equivalence relation which identifies a point $x_{1}$ on the first hypersphere with the point $x_{2}$ on the second hypersphere.

Finally, for $0<E_{GL}<\infty$, the energy level sets are homeomorphic to hyperspheres; hence, we have

(72) $$\Sigma_{E}^{H_{GL}}≃S^{n-1}.$$

**Figure 1:**
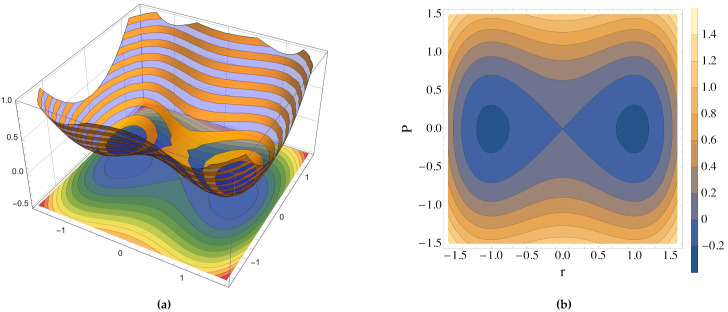
(**a**) Plot of the Hamiltonian function (63). (**b**) Projection of the energy level sets (67) on the P-r plane. In both cases, we used the macroscopic coordinate system (P,r).

In Figure 1, we report a three-dimensional graphic representation of the values of the Hamiltonian function (63) as a function of the macroscopic coordinates (P,r) together with the projection onto the two dimensional plane defined by P-r.

In the case of harmonic oscillators, the energy level sets are always homeomorphic to hyperspheres of radii that depend on the energy values. In fact, by applying the condition (58) to the potential (57), the condition $H_{HO}\left(P,r\right)=E_{HO}$ reduces to the equation of an hypersphere centered in r=P=0 with radius $R\left(E_{HO}\right)=\sqrt{2E_{HO}}$. I.e.,

(73) $$H_{HO}\left(P,r\right)=P^{2}+{\left(\sqrt{\alpha }r\right)}^{2}={\left(\sqrt{2E_{HO}}\right)}^{2}.$$

Thus, we have

(74) $$\Sigma_{E}^{H_{HO}}≃S^{n-1},$$

in the energy range:

(75) E∈[0,∞),

where the lowest energy value is $E_{HO}^{m}=0$.

### 3.2. Geometry of the Energy Level Sets

Let us first report the mathematical expression of the scalar curvature of the energy level sets. The results that we show can be applied to both our cases of interest. Therefore, for simplicity we drop the GL and HO subscripts in the Hamiltonian function.

The fundamental geometric structure is defined by the the unit normal vector field which is

(76) $$\nu =\frac{\nabla H}{∥\nabla H∥}=P-sgn\left(\alpha -\beta r^{2}\right)\frac{q}{r},$$

where we introduced the canonical basis ${\left\{a_{i}\right\}}_{i=1}^{2N}\subset R^{2N}$ so that $q=\sum_{i=1}^{N}q^{i}\;a_{i}$ and $P=\sum_{i=N+1}^{2N}p^{i}a_{i}$, and sgn is the sign function and where the Einstein’s convention on the repeated indices has been adopted.

The trace of Weingarten operator (see Equation (31)) can be immediately computed, and it is

(77) $$Tr\left[W_{\nu }\right]=div\left(\nu \right)=\frac{Lap\;H}{∥\nabla H∥}-\frac{\langle \nabla H,Hess\;H\;\nabla H\rangle }{{∥\nabla H∥}^{3}},$$

where LapH and HessH are, respectively, the Laplacian and the Hessian of the Hamiltonian H with respect to the basis in $R^{N}$. We note that we are using the Hessian as a linear application $Hess\;H:R^{N}⟶R^{N}$.

The trace of the squared Weingarten operator is

(78) $$Tr\left[W_{\nu }^{2}\right]=\frac{Tr[{\left(Hess\;H\right)}^{2}]}{{∥\nabla H∥}^{2}}+\frac{{\langle \nabla H,Hess\;H\;\nabla H\rangle }^{2}}{{∥\nabla H∥}^{6}}-2\frac{{∥Hess\;H\;\nabla H∥}^{2}}{{∥\nabla H∥}^{4}}.$$

Hence, the scalar curvature of the energy level set, given by the relation in Equation (38), is:

(79) $$\begin{array}{c} R\left(\Sigma_{E}^{H}\right)=\frac{{\left(Lap\;H\right)}^{2}}{{∥\nabla H∥}^{2}}-\frac{Tr[{\left(Hess\;H\right)}^{2}]}{{∥\nabla H∥}^{2}}-2\;Lap\;H\frac{\langle \nabla H,Hess\;H\;\nabla H\rangle }{{∥\nabla H∥}^{4}}+2\frac{{∥Hess\;H\;\nabla H∥}^{2}}{{∥\nabla V∥}^{4}}. \end{array}$$

### 3.3. Numerical Results

We now present our results—namely, the solutions of the entropy flow Equation (40)—for the two systems described in the previous section.

In order to do that, we have to: *(i)* solve the Hamilton equations for a suitable set of allowed energy values, depending on the considered potential function—that is, (59) or (57); *(ii)* evaluate the scalar curvature along the dynamics for each energy value in the set of energy above-mentioned; *(iii)* solve the differential Equation (40) or (50).

In practice, we have chosen the following allowed energy subsets $I_{HO}=\left[0,4\right]$ and $I_{GL}=\left[-\alpha^{2}/4\beta ,1\right]$ and we have sampled energy values from these sets with a step $\Delta E=10^{-4}$. Then, we have numerically solved the Hamilton equations with N=150 particles for both potentials (57) and (59) using a second order bilateral symplectic algorithm [36]. We have set α=0.5 and β=0.7 which implies that $\alpha^{2}/4\beta =0.089$ and an integration time step $\Delta t=10^{-4}$. We note that the closer the energy values to the lowest possible, the larger the scalar curvature. In fact, since the energy level sets are always homeomorphic to a sphere (for HO-potential) or two disjoint spheres (for GL-potential with E<0), the level sets shrink to, respectively, a point and two points, being the scalar curvature of a surface roughly given by $R(\Sigma_{E}^{H})\propto 1/E$. Therefore, in order to avoid floating point overflow, we chose as minimum energy values, respectively, $E_{GL}^{m}=-0.08$ and $E_{HO}^{m}=0.1$.

For any energy value E, random initial conditions have been chosen. The normalized geometric integral of the scalar curvature entering the entropy flow equation, namely,

(80) $$\bar{R}\left(e\right)=\frac{\int_{\Sigma_{e}^{H}}R\left(\Sigma_{e}^{H}\right)\;d\sigma_{\Sigma_{e}^{H}}}{vol(\Sigma_{e}^{H})},$$

has been computed along the numerical phase space trajectories under the assumption of ergodicity of the dynamics. The dynamics of the nonlinear, nonintegrable Ginzburg-Landau-like model is chaotic and after the Poincaré-Fermi theorem is ergodic and mixing. To the contrary, the set of uncoupled harmonic oscillators is integrable, however, each single harmonic oscillator is ergodic in its own two-dimensional phase space, and, since all the oscillators have the same frequency, so that they are interchangeable, and the initial conditions are random, also this systems behaves as if it was ergodic, as the stability of the results of the computation of the geometric observables has been checked by changing the initial conditions. In fact, given an observable, Φ, defined on the phase space, the microcanonical averages can be measured along the dynamics as follows [23,34]:

(81) $${\langle \Phi \rangle }_{M}=\frac{\int_{\Sigma_{e}^{H}}\Phi \left(q\right)\;\frac{d\sigma_{\Sigma_{e}^{H}}}{∥\nabla H(q)∥}}{\int_{\Sigma_{e}^{H}}\frac{d\sigma_{\Sigma_{e}^{H}}}{∥\nabla H(q)∥}}\equiv \lim_{T\to \infty }\frac{1}{T}\int_{0}^{T}\Phi \left(q\left(\tau \right)\right)\;d\tau ={\langle \Phi \rangle }_{T}.$$

Hence, in our case, we have:

(82) $$\begin{array}{c} \bar{R}\left(e\right)=\frac{\langle ∥\nabla H∥\;R\left(\Sigma_{e}^{H}\right)\rangle_{M}}{{\langle ∥\nabla H∥\rangle }_{M}}\equiv \frac{\langle ∥\nabla H∥R\left(\Sigma_{e}^{H}\right)\rangle_{T}}{{\langle ∥\nabla H∥\rangle }_{T}}. \end{array}$$

Finally, we solve the entropy flow equation in the version given by Equation (50) using a fourth order Runge–Kutta algorithm [37,38] whose integration step (in energy) is, by construction, inherited by the curvature sampling, namely, $\Delta E=10^{-4}$. The initial condition has been chosen so that $Y(e_{0})=1$ and $Y^{\prime }\left(e_{0}\right)=10^{-3}$.

**Figure 2:**
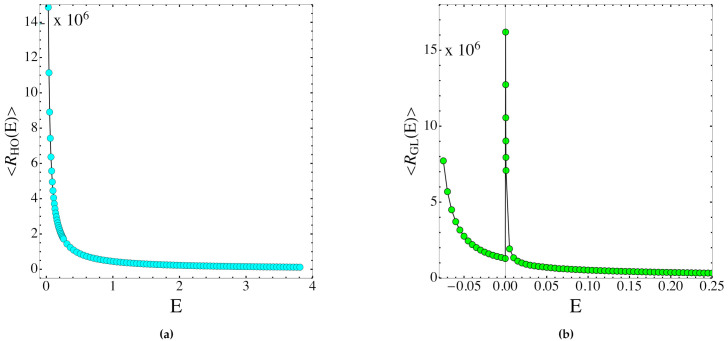
(**a**) Plot of the scalar curvatures of the energy level sets, using the HO system as a function of the energy. (**b**) Plot of the scalar curvatures of the energy level sets using the GL system as a function of the energy. The values of the scalar curvature have been re-scaled by a factor $10^{6}$ for a better visualization.

**Figure 3:**
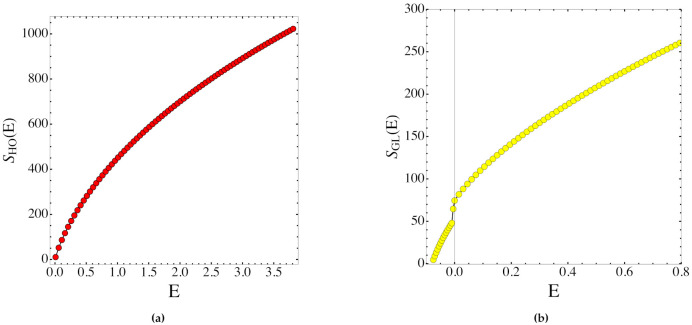
(**a**) Entropy function associated with the harmonic oscillators system. (**b**) Entropy function associated with the system with the GL potential function (57).

We show in Figure 2 and in Figure 3 the scalar curvature and entropy, respectively, for the GL and HO systems.

## 4. Discussion

The theoretical derivation of the entropy function S(e) through the solution of Equation (50)—which involves $\bar{R}\left(e\right)$ in Equation (80)—was done with respect to the energy-like variable e, whereas the numerical simulations were carried out as a function of energy E, and reported in Figure 2 and Figure 3 as functions of E. However, with a large number of degrees of freedom (where large is already the number considered here), e=e(E) is almost a constant parametrization because ∥∇H∥ has small variations for any generic random choice of the initial conditions. In any case, a non-constant deformation between e and E does not hinder the clear-cut qualitative information which is here relevant.

As a matter of fact, the outcomes reported in Figure 2 and Figure 3 show that the entropy is a non-decreasing always concave function of the energy displaying a jump in correspondence of the PT in the GL model. On the other hand, the total scalar curvature displays a non-monotonous, discontinuous pattern in the presence of the PT of the GL model, and this—after Equation (56)—means that some non-trivial change of topology of the energy level sets is behind the PT. As a matter of fact, the outcomes reported in Figure 2 and Figure 3 show that the entropy is a non-decreasing, always concave function of the energy displaying a jump in correspondence of the PT in the GL model. The property of the entropy pattern of being always concave, that is, without turning somewhere from concave to convex, entails the absence of regions of negative specific heat, as expected for the model under consideration with polynomial short-range interactions. On the other hand, the total scalar curvature displays a non-monotonous, discontinuous pattern inthe presence of the PT of the GL model, and this—after Equation (56)—means that some non-trivial change of topology of the energy level sets is behind the PT. What is reported here has nothing to do yet with sufficiency conditions; it is just a consistency check, mainly showing how Equation (56) actually works. As already remarked above, on the basis of this equation, the future steps to define sufficient topological conditions for the appearance of a PT in a physical system will be worked out in the framework of real analysis—that is, identifying some kinds of variation with energy of the Betti numbers of the energy level sets in order to make the solution Y(e) of Equation (56)—and consequently the entropy function S(e)—of a suitably low differentiability class.

## Data Availability

Fortran and Mathematica codes and simulation data are available upon request addressed to L.D.C.
